# Closed-loop chemical recycling of cross-linked polymeric materials based on reversible amidation chemistry

**DOI:** 10.1038/s41467-022-35365-4

**Published:** 2022-12-09

**Authors:** Bo Qin, Siyuan Liu, Zehuan Huang, Lingda Zeng, Jiang-Fei Xu, Xi Zhang

**Affiliations:** 1 Key Lab of Organic Optoelectronics and Molecular Engineering, Department of Chemistry, Tsinghua University, Beijing, 100084 P. R. China; 2 Melville Laboratory for Polymer Synthesis, Yusuf Hamied Department of Chemistry, University of Cambridge, Cambridge, CB2 1EW UK

**Keywords:** Polymers, Polymers, Polymer synthesis

## Abstract

Closed-loop chemical recycling provides a solution to the end-of-use problem of synthetic polymers. However, it remains a major challenge to design dynamic bonds, capable of effective bonding and reversible cleaving, for preparing chemically recyclable cross-linked polymers. Herein, we report a dynamic maleic acid tertiary amide bond based upon reversible amidation reaction between maleic anhydrides and secondary amines. This dynamic bond allows for the construction of polymer networks with tailorable and robust mechanical properties, covering strong elastomers with a tensile strength of 22.3 MPa and rigid plastics with a yield strength of 38.3 MPa. Impressively, these robust polymeric materials can be completely depolymerized in an acidic aqueous solution at ambient temperature, leading to efficient monomer recovery with >94% separation yields. Meanwhile, the recovered monomers can be used to remanufacture cross-linked polymeric materials without losing their original mechanical performance. This work unveils a general approach to design polymer networks with tunable mechanical performance and closed-loop recyclability, which will open a new avenue for sustainable polymeric materials.

## Abstract (web summary)

Closed-loop chemical recycling provides a solution to the end-of-use problem of synthetic polymers but the design of dynamic bonds for preparing chemically recyclable cross-linked polymers remains challenging. Here, the authors report a dynamic reversible amidation reaction between maleic anhydrides and secondary amines.

## Introduction

Cross-linked polymers possess outstanding mechanical properties, structural stability and solvent resistance, owing to their covalent network structures^1^. Such permanently cross-linked networks are difficult to break even at high temperature or dissolve in common solvents^2,3^; thus, recycling cross-linked polymers is particularly impractical. As a result, most cross-linked polymeric materials have to be discarded after usage, causing a major problem in the sustainable polymer economy. To address this problem, incorporation of dynamic bonds into cross-linked polymers have enabled them to be reshaped and reprocessed through bond exchange^4–23^. However, the thermal reprocessing at the required high temperature usually poses a risk of the thermo-degradation of polymers, which may lead to the decrease of their mechanical properties^24,25^. Alternatively, chemical recycling at ambient temperature is more facile to depolymerize plastic wastes into soluble oligomers or even monomers for reproducing new polymers^26–39^. Therefore, closed-loop chemical recycling through transforming cross-linked polymers into monomeric feedstock under ambient conditions exhibits great potential to completely recover or even improve the mechanical properties of the regenerated polymers^40^, which will play an important role in the circular polymer economy.

Over the past decade, many efforts have been devoted by polymer chemists to employ various chemical bonds, including ester bonds^41–47^, thioester bonds^48^, ether bonds^49^, carbonate bonds^43^, olefinic bonds^50^, diketoenamine bonds^51^, disulfide bonds^52–55^, boroxine bonds^56,57^, etc. in the construction of polymeric materials with closed-loop recyclability. Reversible bond reorganization of the above covalent bonds has enabled the regeneration of polymers, which are inherently depolymerizable to form pristine monomers under certain conditions. The breakage of covalent bonds within polymeric networks often requires an energy-intensive process and undergoes side reactions, resulting in mixed products rather than clean reusable monomers^58,59^. Although the recovered monomers can be used to further prepare new polymeric materials, the subsequent polymerization usually depends on highly efficient catalysis as well. Therefore, rational design of dynamic covalent bonds, capable of effective bonding and reversible cleaving both under mild yet orthogonal conditions, is prerequisite for the fabrication of polymer networks with closed-loop recyclability.

**Fig. 1:**
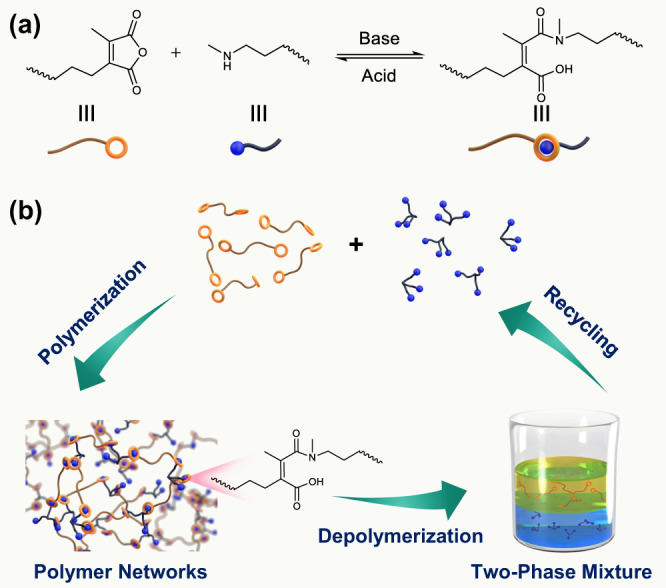
Design and closed-loop chemical recycling of cross-linked polymeric materials. Schematic of (**a**) maleic acid tertiary amide bonds constructed from maleic anhydrides and secondary amines; and (**b**) polymer networks prepared from bifunctional maleic anhydrides and multifunctional secondary amines and their depolymerization in acid aqueous solution. Two monomers can be separated and regained in a closed-looped recycling process.

Herein, we introduce a bonding system of reversible amidation chemistry, based upon controllable reaction between maleic anhydrides and secondary amines, for constructing a series of polymer networks with chemical recyclability (Fig. 1a). Highly efficient and reversible reactions of maleic anhydrides and primary amines have been widely exploited to prepare acid-labile linkages and caging groups within biomedical materials^60–65^. However, the resultant secondary amide and carboxylic groups can undergo a subsequent dehydration to generate a stable imide structure at elevated temperatures, which has limited their use in the design of recyclable polymeric materials. To circumvent such irreversible dehydration, introducing secondary amines as the substrate instead of primary ones can avoid this imidation, thus developing a type of reversible amidation to form dynamic maleic acid tertiary amide bonds. On this basis, a series of closed-loop recyclable cross-linked polyamic acid plastics and elastomers were readily fabricated through polymerization of ditopic maleic anhydride monomers and multifunctional secondary amines. On one hand, these polymeric materials could exhibit remarkable mechanical properties under the service condition. On the other hand, these polymer networks could be mildly and completely depolymerized in an acidic aqueous solution (pH ~ 0) at ambient temperature. Notably, rational design of maleic anhydride and secondary amine monomers with distinct solubilities in acidic aqueous solution allows for the recovery of monomers with high purity and yield from the depolymerized mixtures (Fig. 1b). Meanwhile, the recovered monomers can be used to remanufacture cross-linked polymeric materials for several times without losing their original mechanical performance. Therefore, a set of polymer networks with tailor-made structures and properties could be constructed and recycled without losing their values.

## Results

### Study on the reversible amidation reaction

**Fig. 2:**
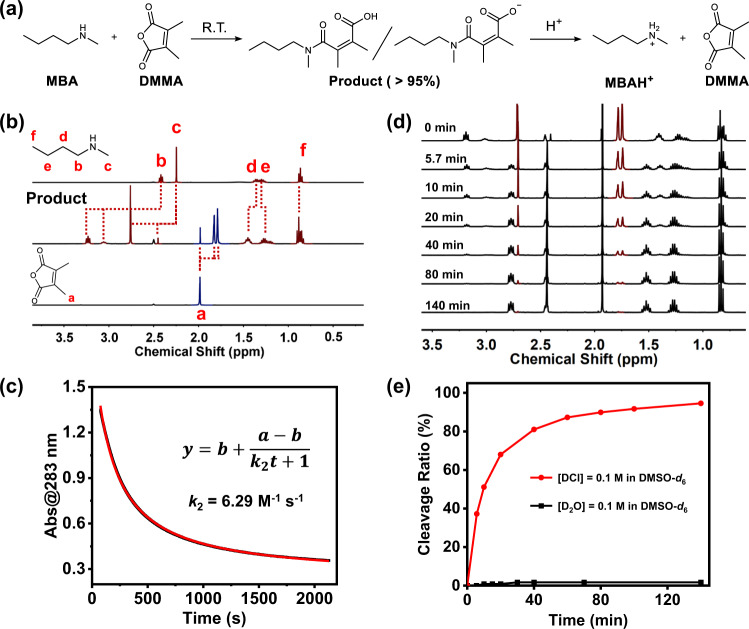
Characterization of the reversible amidation reaction. **a** Schematic of the model reaction between MBA and DMMA and the cleavage of the addition product. **b** ^1^H NMR spectra of MBA (top), the product (middle), and DMMA (bottom) at the concentrations of 0.1 M. **c** Diagram of Abs@283 versus reaction time of 1.0 mM MBA and DMMA solutions at 20 °C and the fitting curve (red). **d** ^1^H NMR spectra of the product in DMSO-*d*_6_ with varied time after adding DCl/D_2_O at the concentration of 0.1 M. **e** Plot of the cleavage ratio versus the reaction time with 0.1 M DCl and D_2_O in DMSO-*d*_6_, respectively.

To investigate the anhydride-amine reaction, two model molecules, dimethylmaleic anhydride (DMMA) and N-methylbutyl amine (MBA), were firstly designed (Fig. 2a). DMMA and MBA were equimolarly mixed at 0.1 M in DMSO-*d*_6_ and the obtained mixture was reacted for 5 min at room temperature. As shown in Fig. 2b, the decrease of the proton peaks from DMMA (methyl, *δ* = 1.98 ppm) and MBA (methyl, *δ* = 2.44 ppm) together with the appearance of new peaks (*δ* = 1.79 and 1.82 ppm) ascribed to the addition product suggests a successful reaction between anhydride and secondary amine at ambient temperature. Integrating the peaks ascribed to the addition product provides the yield to be >95% within 5 min at room temperature, indicative of an effective conversion, while the purity of the product was also indicated by the ^13^C NMR (Supplementary Fig. 1) and the ESI-MS spectrum (Supplementary Fig. 2). All the above data confirms the effective addition reaction between DMMA and MBA.

To understand the in-depth reaction kinetics, time-dependent UV-vis experiments were implemented to determine the reaction rate. The absorption change at 283 nm (Abs@283 nm) was used to monitor the reaction kinetics between DMMA and MBA. This reaction is identified as a pseudo first-order reaction for DMMA in the presence of 20 eq. of MBA (Supplementary Fig. 3), and the second-order reaction rate constant for equimolar DMMA and MBA is determined to be 6.29 M^−1^ s^−1^ at 20 °C (Fig. 2c). Clearly, such amidation between maleic anhydride and secondary amine is efficient and rapid.

We then wondered whether the formed maleic acid tertiary amide bond could be cleaved under acidic conditions. To answer this question, an aqueous solution of DCl was added into the above amidation product in DMSO-*d*_6_ to facilitate the cleavage reaction (Fig. 2a), and the concentration of DCl was fixed at 0.1 M. Extensive time-dependent ^1^H NMR experiments were carried out to monitor the cleavage process. As shown in Fig. 2d, the proton peak at *δ* = 2.76 ppm, belonging to the methyl protons attached to the amide nitrogen, as well as another two peaks at *δ* = 1.79 and 1.82 ppm, belonging to the methyl protons next to the double bond in the product, gradually disappear as the cleavage reaction progresses. By integrating these peaks, almost all of the addition product (>99%) are cleaved into the original anhydride and protonated amine reactants within 140 min. As a control, in the presence of D_2_O under neutral condition, no significant hydrolysis is detected by ^1^H NMR (Fig. 2e, Supplementary Fig. 4 and Supplementary Fig. 5) with <2% yield after 140 min. The above cleavage reaction could also effectively occur under low temperature, where >94% of the addition product can be cleaved under acidic condition at 0 °C within 320 min (Supplementary Figs. 6–8). These results indicate that the maleic acid tertiary amide bond is readily cleavable under mild acidic environment to recover the original anhydride and secondary amine.

### Design and preparation of the elastomeric network BMA-TMEN

**Fig. 3:**
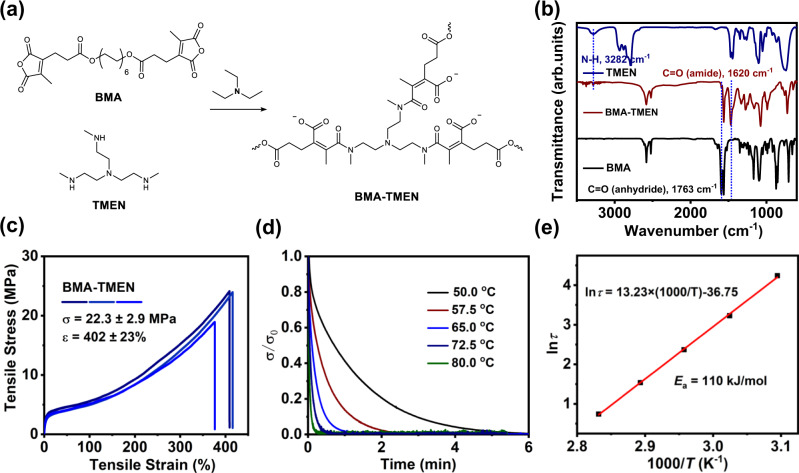
Preparation and characterization of rubbery BMA-TMEN. **a** Schematic of the polymer network BMA-TMEN synthesized by the polycondensation of TMEN and BMA in the presence of triethylamine. **b** FT-IR spectra of TMEN, BMA-TMEN and BMA. **c** Stress-strain curve of BMA-TMEN. **d** Normalized stress relaxation curves of BMA-TMEN. **e** Arrhenius plot of the characteristic relaxation time against inverse temperature.

Having confirmed the reversible amidation between maleic anhydride and secondary amine, we further utilized such dynamic maleic acid tertiary amide bond to construct cross-linked polymer networks with potential chemical recyclability. To this end, a bifunctional maleic anhydride (BMA) and a trifunctional secondary amine named as tris(2-methylaminoethyl)amine (TMEN) were designed and used as the monomers (Fig. 3a, Supplementary Figs. 9–12). To fabricate the polymer network BMA-TMEN, two chloroform solutions of BMA (0.15 M, 60 mL) and TMEN (0.20 M, 30 mL) were mixed and added with 0.018 mol triethylamine, thus immediately forming a highly viscous liquid. Notably, dichloromethane and ethyl acetate can also be used as the solvent. Upon the evaporation of the resultant mixture, a homogeneous and yellow film of BMA-TMEN was obtained (Supplementary Figs. 14 and 15) and further characterized by FT-IR. Figure 3b and Supplementary Fig. 16 showed that the vibration peaks at 1763 cm^−1^ (anhydride ring) and 3282 cm^−1^ (secondary amine group) completely disappeared in the IR spectrum of BMA-TMEN, suggesting the high monomer conversion. Meanwhile, a new peak at 1620 cm^−1^ ascribed to the tertiary amide bond appears, further indicating the formation of cross-linked polymers. This result indicates that a type of cross-linked polyamic acid network is successfully constructed through the highly efficient amidation reaction.

### Thermal and mechanical characterization of BMA-TMEN elastomers

To characterize the thermal behaviors of the resultant polyamic acid networks, dynamic mechanical analysis (DMA) and differential scanning calorimetry (DSC) were employed. The glass transition temperature (*T*_g_) of BMA-TMEN was measured. As shown in Supplementary Fig. 17, the *T*_g_ determined by DSC is around 8 °C, which is relatively lower than that measured by DMA (19 °C) (Supplementary Fig. 18). This DMA curve further shows that the BMA-TMEN network exhibits clear rubbery plateaus in the storage modulus curve above the *T*_g_, confirming the formation of cross-linked networks.

To further investigate the mechanical properties of BMA-TMEN, uniaxial tensile tests were carried out at an extension rate of 10 mm min^−1^ under ambient conditions. As shown in Fig. 3c, BMA-TMEN displays a stress at break up to 22.3 MPa with a maximum elongation up to 402%. Based upon integration of stress-train curves, the toughness of the BMA-TMEN network is calculated to be around 40.5 MJ m^−3^. These results confirm that this polyamic acid network, fabricated through the polycondensation of maleic anhydride and secondary amine monomers, indeed exhibits remarkable stretchability, high mechanical strength and toughness.

Dynamic behavior of the obtained polymer network was studied by a series of temperature-dependent stress relaxation experiments. As shown in Fig. 3d, all of these samples underwent rapid reconfiguration and complete stress relaxation at elevated temperatures, where higher temperatures promote faster relaxation. The plot of the characteristic relaxation time values unambiguously follows a typical Arrhenius relationship (Fig. 3e) with a calculated apparent activation energy (*E*_a_) of 110 kJ mol^−1^ for bond exchange in the solid state. All of the results indicate that the network structure of cross-linked polymeric materials can be readily rearranged through dynamic exchange among the maleic acid tertiary amide bonds.

### Chemical recycling of BMA-TMEN elastomers

**Fig. 4:**
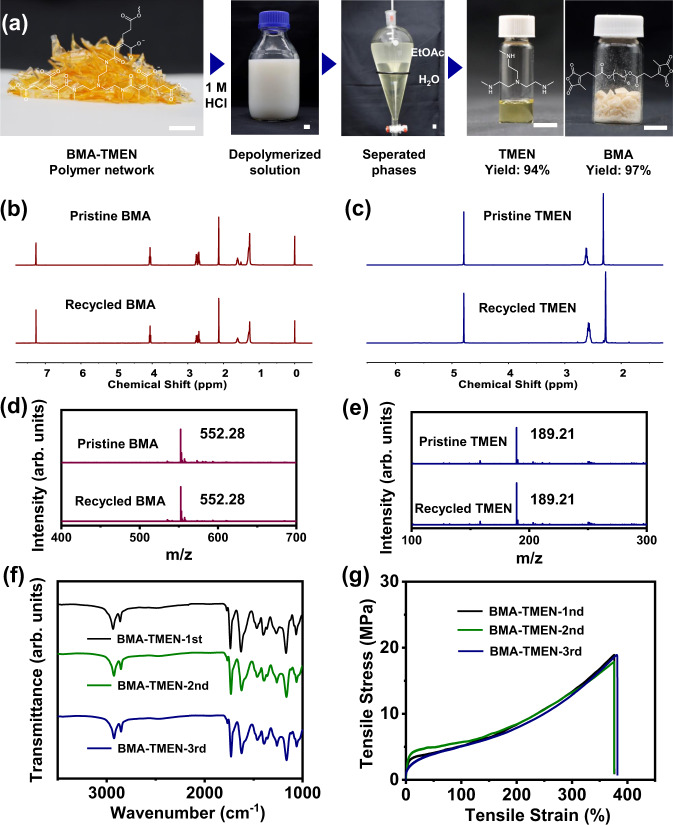
Chemical recycling and repolymerization of BMA-TMEN. **a** Photographs of the depolymerization of polymer networks, the separation and recycling procedures of BMA and TMEN monomers by the liquid-liquid extraction method (scale bar: 1 cm). **b** ^1^H NMR spectra of pristine and recycled BMA. **c** ^1^H NMR spectra of pristine and recycled TMEN. **d** ESI-MS spectra of pristine and recycled BMA. **e** ESI-MS spectra of pristine and recycled TMEN. **f** FT-IR spectra of BMA-TMEN and repolymerized BMA-TMEN from recycled monomers. **g** Stress-strain curves of BMA-TMEN and repolymerized BMA-TMEN from recycled monomers.

After elucidating robust and dynamic nature of the obtained polymer networks, we further wondered whether these networks could be chemically recycled under acidic conditions and the corresponding monomers could be recovered through separation and purification from the depolymerization mixtures. As a proof of concept, several sheets of polymer samples were firstly cut into small pieces and then immersed into an aqueous solution of 0.1 M HCl (Supplementary Fig. 19). It took around 1 h to dissolve all the samples under stirring at room temperature. By increasing the concentration of HCl to 1 M, all the specimens were completely dissolved within 30 min, and the solution phase became a steady emulsion without observable residue solids, indicating the complete depolymerization under the acidic condition (Fig. 4a). Subsequently, the water-soluble protonated secondary amine monomers and oil-soluble anhydride monomers in the above emulsion were separated through a simple liquid-liquid extraction between the water phase and ethyl acetate phase. After evaporation of the remaining ethyl acetate, the anhydride monomer BMA can be directly recovered without further purification, and the amine monomer TMEN can be also readily regenerated through the deprotonation on basic resins.

^1^H NMR and ESI-MS experiments were performed to evaluate the purity of the recycled monomers. The obtained ^1^H NMR (Fig. 4b, c) as well as the ESI-MS (Fig. 4d–e) spectra of recycled anhydride and secondary amine are almost identical to those of the pristine ones, confirming the high purity of the recycled monomers. Notably, the isolation yields of the anhydride and secondary amine reached up to 97 and 94%, respectively, indicative of their high efficiency of monomer recovery. Meanwhile, the recycled monomers with high purity were further repolymerized to construct the reborn BMA-TMEN networks (Supplementary Fig. 20). Figure 4f shows that the FT-IR spectra of the repolymerized BMA-TMEN networks (2nd and 3rd) are almost identical to that of the original one (1st), which indicates that no significant changes of structures are observed within the polymer networks during multiple chemical recycling processes. In addition, Fig. 4g and Supplementary Fig. 21 show that the tensile and DSC curves of the repolymerized BMA-TMEN networks (2nd and 3rd) overlap with those of the original network (1st). This result indicates that their mechanical and thermal properties are completely recovered over two continuous chemical recycling cycles. Subsequently, another round of recycling again results in pure monomers obtained in high yields (Supplementary Figs. 22–24). All of the above results confirm the excellent closed-loop recyclability of the BMA-TMEN elastomer.

**Fig. 5:**
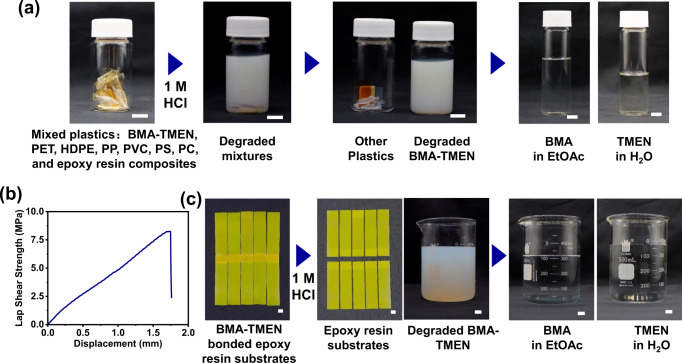
Selective chemical recycling of BMA-TMEN. **a** Depolymerization and recycling process of BMA-TMEN from the mixed plastic wastes containing PET, HDPE, PP, PVC, PS, PC and epoxy resin composites (scale bar: 1 cm). **b** Lap shear strength-displacement curve of BMA-TMEN in bonding epoxy resin substrates. **c** Depolymerization and recycling process of BMA-TMEN from the bonded epoxy resin substrates.

Selective monomer recycling from mixed plastic waste streams is of great importance in the recycling process of recyclable materials. To demonstrate this point, we further carried out a model experiment to recycle BMA-TMEN networks from a mixture of common plastics, including polyethylene terephthalate (PET), high-density polyethylene (HDPE), polypropylene (PP), polyvinyl chloride (PVC), polystyrene (PS), polycarbonate (PC) and epoxy resin composites. Figure 5a shows that the mild recycling condition (1 M HCl) used in our research cannot degrade any of the above common plastics, which clearly indicates the selective depolymerization, separation and recovery of BMA-TMEN networks from plastic waste streams.

Furthermore, the abundant carboxylic groups in the BMA-TMEN network enable it to be applied as adhesive materials. Lap shear tests were performed to investigate the adhesive performance of the BMA-TMEN network. Figure 5b shows that BMA-TMEN possesses a remarkable adhesive capability to epoxy resin substrates with a shear strength up to 8.50 ± 0.80 MPa. Impressively, this BMA-TMEN adhesive can also be completely depolymerized and chemically recycled from the bonded epoxy resin substrates through immersing in 1 M HCl for 30 min (Fig. 5c). In addition, no clear damage is observed on the epoxy resin substrates after the recycling process. All of the results elucidate that this type of cross-linked polymeric material indeed exhibits highly-selective chemical recyclability to recover monomer feedstocks from mixed commodity plastics.

### Construction of cross-linked polymeric materials LPEI-BMA_*x*_ spanning from rubbery to glassy states

**Fig. 6:**
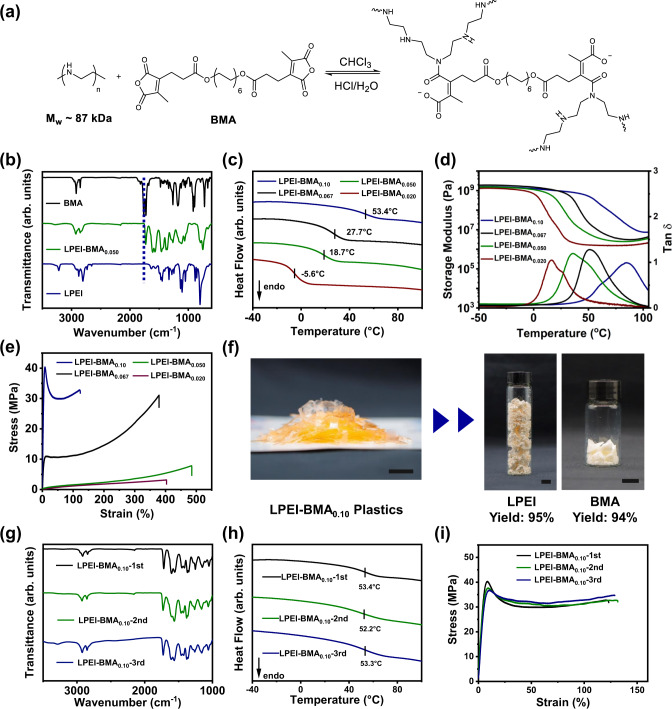
Preparation and Characterization of LPEI-BMA_*x*_. **a** Schematic of the polymer network LPEI-BMA synthesized by the side-chain cross-linking and its depolymerization under the acidic condition. **b** FT-IR spectra of the cross-linker BMA, polymer network LPEI-BMA_0.050_, and LPEI. **c** DSC curves, **d** DMA curves, and (**e**) representative stress-strain curves of LPEI-BMA_*x*_ samples with varied molar ratio *x* between the cross-linkers and ethylene imine units. **f** Photographs of chemical recycling of the polymer network LPEI-BMA_0.10_ into the feedstocks LPEI and BMA (scale bar: 1 cm). **g** FT-IR spectra, **h** DSC curves, and **i** stress-strain curves of LPEI-BMA_0.10_ and repolymerized LPEI-BMA_0.10_ from recycled monomers.

To further extend the rubbery cross-linked networks to the glassy plastic materials, another strategy of side-chain cross-linking was applied to construct a set of cross-linked polymeric materials. A commercially available polyamine, linear polyethyleneimine (LPEI, *M*_w_ ~ 87000 g/mol) with abundant secondary amine motifs, was chosen as the polymer backbone and used to be cross-linked by the above bifunctional anhydride monomer BMA (Fig. 6a, Supplementary Figs. 25 and 26). It is noteworthy that no additional base is needed to catalyze the reaction because the excess of secondary amine groups on the LPEI can behave as the base itself. After heating at 60 °C in chloroform along with solvent evaporation, homogeneous polymeric films were obtained (Supplementary Fig. 27) and then characterized by FT-IR. As shown in Fig. 6b and Supplementary Fig. 28, no peak ascribed to the anhydride ring was observed, indicating the high conversion of BMA and the successful preparation of LPEI-BMA network.

To understand the effect of cross-linker ratio on the mechanical properties of LPEI-BMA networks, we prepared a series of samples LPEI-BMA_*x*_, where *x* referred to the molar ratio between anhydride cross-linkers and ethylene imine units. As shown in the DSC curves (Fig. 6c), tuning *x* from 0.020 to 0.10 leads to a remarkable increase in *T*_g_ values from −5.6 °C to 53.4 °C, suggesting that the flexibility of polymer chains is dramatically decreased alongside the increase of the degree of cross-linking. Notably, as shown in Fig. 6d, all the samples show rubbery plateaus in the storage moduli above *T*_g_, indicating the formation of cross-linked structures. Such wide range of *T*_g_ values also confirms that the mechanical properties of cross-linked polymeric materials can be well-tuned from rubbery to glassy states.

Furthermore, tensile tests were performed to investigate the mechanical properties of LPEI-BMA_*x*_. As summarized in Fig. 6e and Supplementary Table 1, increasing *x* from 0.020 to 0.050, 0.067, and 0.10 within the LPEI-BMA_*x*_ networks resulted in a significant increase in the stress at break from 3.0 to 8.2, 31.9, and 32.1 MPa, respectively. Correspondingly, the Young’s modulus of LPEI-BMA_*x*_ can be well modulated over nearly three orders of magnitude from 2.5 MPa to 0.88 GPa. Impressively, LPEI-BMA_0.10_ displays a high yield strength of 38.3 MPa, indicating its mechanical robustness as cross-linked plastics. Therefore, this represents a tailorable family of cross-linked polyamic acid materials with remarkable mechanical properties spanning from soft elastomers to rigid plastics.

### Chemical recycling of the cross-linked plastic LPEI-BMA_0.10_

Chemical recyclability of the glassy polyamic acid material was further studied through acid treatment. Taking LPEI-BMA_0.10_ plastic as an example, the polymer specimens were cut into small pieces and then immersed into an aqueous solution of HCl. Under stirring at room temperature, all the samples were fully dissolved in an aqueous solution of 2.0 M HCl within 45 min, while it took around 6 h for dissolving all the samples with 0.1 M HCl (Supplementary Figs. 29–31). Through a simple extraction by ethyl acetate, BMA was regenerated with a high isolation yield of 94%, and 95% of LPEI was also readily recovered through recrystallization after tuning the pH from 0 to 10 (Fig. 6f). Indicated by ^1^H NMR analysis (Supplementary Figs. 32 and 33), both BMA and LPEI were successfully recycled with high purity. Furthermore, the recycled monomers were used for the reconstruction of LPEI-BMA_0.10_ plastics (Supplementary Fig. 34). Figure 6g–i show that the FT-IR spectra, DSC curves and tensile curves of repolymerized LPEI-BMA_0.10_ plastics from recovered monomers are almost identical to those of the original one, revealing that the reborn plastics maintain high structural integrity and mechanical properties. Therefore, this tailorable family of polyamic acid materials constructed through the side-chain cross-linking strategy displays excellent chemical recyclability to regenerate original feedstocks with high purity and efficiency.

## Discussion

In summary, we have successfully developed a type of chemically recyclable cross-linked polyamic acid materials through incorporation of dynamic maleic acid tertiary amide bonds. This advanced dynamic bond based upon a highly efficient amidation between maleic anhydride and secondary amine, enabling reversible chemical control over depolymerization of the resultant polymer networks. Closed-loop chemical recycling is achieved through depolymerization in acidic aqueous solutions and simple two-phase separation to regenerate monomers with high quality and efficiency, and the recovered monomers are used to remanufacture cross-linked polymeric materials without losing their original mechanical performance. Notably, the mild recycling conditions allow highly selective depolymerization of the cross-linked networks and recovery of the monomers from mixed plastic waste streams. Impressively, a library of cross-linked polyamic acid materials with remarkable mechanical properties, spanning from strong elastomers to rigid plastics, are rationally devised and constructed through two efficient strategies, including polycondensation and side-chain cross-linking. We envision that this line of research will pave a new avenue for the design and construction of next-generation polymeric materials with closed-loop chemical recyclability, serving the sustainable polymer economy.

## Methods

### Preparation of the polymer network BMA-TMEN

Typically, BMA (4.81 g, 0.009 mol) was dissolved in anhydrous CHCl_3_ (60 mL), and TMEN (1.13 g, 0.006 mol) was dissolved in anhydrous CHCl_3_ (30 mL). Then, the solution of BMA was quickly mixed into the solution of TMEN under stirring. Triethylamine (2.50 mL, 0.018 mol) was slowly added into the above mixture. After stirring for 5 min, the viscous mixture was poured into a PTFE mold, and a homogeneous and yellow film was obtained by solvent evaporation.

### Chemical recycling of the BMA-TMEN elastomers

In a typical experiment, the BMA-TMEN film was previously synthesized via the polycondensation of BMA (5.03 g) and TMEN (1.18 g) in the presence of triethylamine (2.61 mL). Then, all the films were cut into small pieces, and then immersed in a 1 M aqueous HCl solution (450 mL). After stirring 30 min, the formed steady emulsion was extracted with ethyl acetate (130 mL × 3). Next, the organic phase and water phase were separated, and the organic phase was filtered and dried by Na_2_SO_4_. After evaporating organic solvents, the anhydride monomer BMA (4.87 g) could be directly recovered without further purification steps. Meanwhile, the amine monomer TMEN (1.11 g) was also obtained through the deprotonation reaction via a regenerative resin-based process according to the reported procedures^50^. Therefore, the yield of BMA and TMEN was 97 and 94%, respectively.

The above mild recycling condition cannot degrade common plastics, thus allowing for selective recovery of the polymeric materials BMA-TMEN from plastic waste streams. Through mixing BMA-TMEN and other common plastics, including PET, HDPE, PP, PVC, PC, PS and epoxy resin composite, only BMA-TMEN could be depolymerized within 30 min in a 1 M aqueous HCl solution. The residual solids were readily separated and the monomers, including BMA and TMEN, were recovered through the above recycling method.

### Preparation of the polymer networks LPEI-BMA_*x*_

Using the synthesis of LPEI-BMA_0.10_ as a demonstration, the fresh LPEI (1.50 g, M_w_ ~ 87 kDa) was dissolved in anhydrous CHCl_3_ (150 mL) and BMA (1.86 g) was dissolved in anhydrous CHCl_3_ (15 mL). Then, the solution of BMA was quickly added into the solution of LPEI under stirring, which was then rapidly poured into a PTFE mold. A homogeneous and yellow film was obtained by solvent evaporation. LPEI-BMA_0.067_, LPEI-BMA_0.050_, LPEI-BMA_0.020_ were prepared through the similar procedures by changing the molar ratio of BMA and ethyleneimine units.

### Chemical recycling of the glassy LPEI-BMA_0.10_

In a typical experiment, the LPEI-BMA_0.10_ film was synthesized via the polymerization of 1.86 g BMA and 1.55 g LPEI. After the tensile measurements, all the films were cut into small pieces, and then immersed in a 2 M aqueous HCl solution (400 mL). After stirring 45 min, the solution was cooled in liquid nitrogen for 30 s, producing abundant white precipitation of BMA and protonated LPEI. Through the extraction with ethyl acetate (400 mL), BMA (1.74 g) was directly obtained after evaporating organic solvents. Meanwhile, LPEI (1.47 g) could be recovered through recrystallization after tuning the pH to 10, which was similar with the synthetic route of LPEI from Poly(2-ethyl-2-oxazoline). Therefore, the isolated yield of BMA and LPEI was 94 and 95%, respectively.

## Supplementary information

Supplementary Information

## Data Availability

The data that support the findings of this study are available from the corresponding author upon request.
