# Supplementary material for: Closed-loop chemical recycling of cross-linked polymeric materials based on reversible amidation chemistry
Source: Nat Commun. 2022 Dec 9;13:7595. doi: 10.1038/s41467-022-35365-4 (PMC9734120; doi:10.1038/s41467-022-35365-4)
Supplement: Supplementary file 1 — Supplementary Information [file 41467_2022_35365_MOESM1_ESM.pdf]

Supplementary Information

Closed-loop chemical recycling of cross-linked polymeric materials based on reversible amidation chemistry

Bo Qin[1, 3], Siyuan Liu[1, 3], Zehuan Huang[2], Lingda Zeng[1], Jiang-Fei Xu[1], and Xi Zhang[1]*

[1] Key Lab of Organic Optoelectronics and Molecular Engineering, Department of Chemistry, Tsinghua University, Beijing, 100084, China

[2] Melville Laboratory for Polymer Synthesis, Yusuf Hamied Department of Chemistry, University of Cambridge, Cambridge CB2 1EW, U.K

* E-mail: xujf@mail.tsinghua.edu.cn (Jiang-Fei Xu)

[3] These authors contributed equally to this work.

Table of Contents

1. Materials and Instrumentation
2. Characterization of the amidation reaction between dimethylmaleic anhydride (DMMA) and N-methylbutyl amine (MBA)
3. Synthesis and Characterization of Bifunctional Maleic Anhydride Monomer (BMA)
4. Characterization of the Amidation Reaction between BMA and Bifunctional Secondary Amines
5. Synthesis and Characterization of Polyamic Acid Networks BMA-TMEN
6. Chemical Recycling and Reconstruction of Polyamic Acid Networks BMA-TMEN
7. Synthesis and Characterization of Linear Polyethyleneimine (LPEI)
8. Synthesis and Characterization of Polyamic Acid Networks LPEI-BMA$_{0.10}$
9. Chemical Recycling and Reconstruction of Polyamic Acid Networks LPEI-BMA$_x$
10. Supplementary References

1. Materials and Instrumentation

Materials. 3,4-dimethylfuran-2,5-dione (DMMA, 98%), sodium hydride (60%, dispersion in mineral oil), calcium hydride (95%), triethylamine (99.5%), anhydrous pyridine (99.5%), DMSO (99%), THF (99%), and CH_2Cl_2 (99%) were purchased from J&K Scientific, China. Dimethyl 2-Oxoglutarate (95%), 1,12-Dodecanediol (98%) and triethyl 2-phosphonopropionate (98%) were purchased from Meryer (Shanghai) Chemical Technology, China. Tris[2-(methylamino)ethyl]amine (TMEN, 97%) and poly(2-ethyl-2-oxazoline) (M_w ~ 200 kDa, PDI 3-4) were purchased from Sigma-Aldrich. N-methylbutan-1-amine (MBA, 93%) were purchased from Tokyo Chemical Industry Co., Ltd, Japan. The ion-exchange resin Amberlyst A-26(OH) was purchased from Acros and washed 5 times prior to use. Anhydrous $CHCl_3$ was obtained through the reflux with CaH_2 for more than 24 h. All chemical compounds were used without further purification.

Instrumentation. ^1H and ^{13}C NMR spectra were recorded on a JEOL JNM-ECA400 spectrometer at 400 MHz and 100 MHz, respectively. FT-IR spectra were recorded on an UATR Two FT-IR spectrometer by using an ATR accessory between 600 and 4000 cm^{-1}. ESI-MS was recorded on a LTQ LC/MS apparatus. UV-vis spectra were performed on a HITACHI U-3010 spectrophotometer. DSC curves were recorded on a TA instrument Discovery 250 system with a flow N_2 rate of 50 mL/min. All the samples (5-10 mg) were heated in an arranged temperature range with a rate of 10 °C/min. To eliminate the thermal history, data from the second heating cycle were used to determine the glass transition temperatures. DMA measurements were performed on a TA instrument Discovery 850 in a tensile mode. The rectangular samples [ca. 6 mm (L)×5 mm (W)× 0.3 mm (T)] were measured under an oscillatory strain by heating in an arranged temperature range at a heating rate of 3 °C/min and a frequency of 1 Hz. Stress-relaxation experiments were performed on a Discovery 850 in a tensile mode, in which the samples were stretched under a constant deformation of 10% at different temperatures. The stress was monitored as a function of time until at least 37% of initial stress was reached. Tensile testing was measured by using a universal testing machine (INSTRON 6800) at room temperature. For the tensile test, all the samples were cut into Type IV standard shapes. The gauge length and width of dumbbell-shaped films was 10 mm and 2 mm, respectively. The thickness of the films was around 0.1 mm. The rate of extension was fixed at

10 mm min^{-1} for tensile tests. Meanwhile, Young's modulus values were calculated by fitting the slope of the stress-strain curve in the initial linear region. The toughness is defined as the area surrounded by the stress-strain curves.

2. Characterization of the amidation reaction between dimethylmaleic anhydride (DMMA) and N-methylbutyl amine (MBA)

2.1 ^{13}C NMR spectrum and ESI-MS spectrum of the product of the reaction between DMMA and MBA

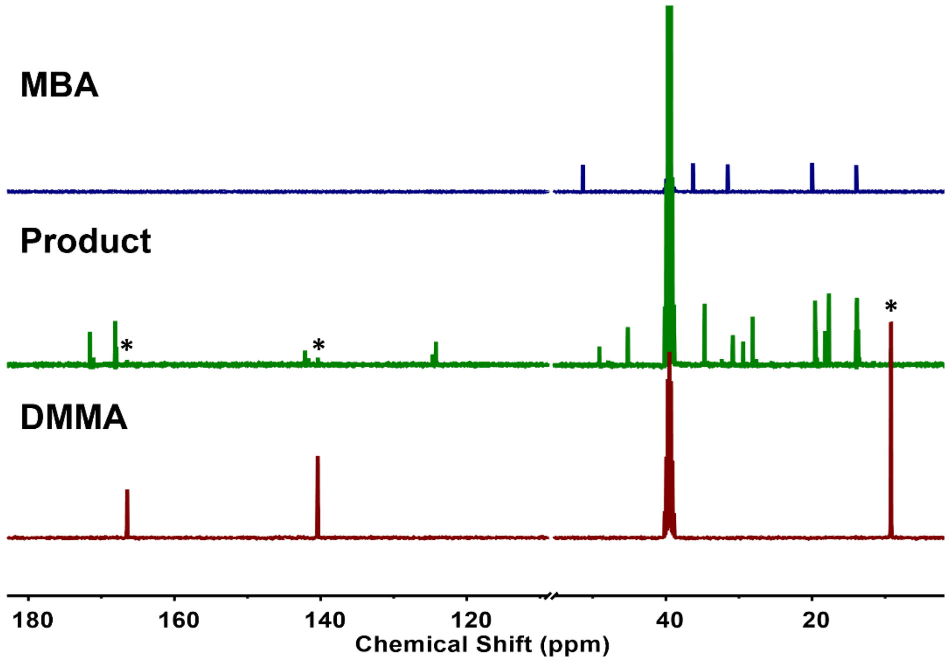

Supplementary Figure 1. ^{13}C NMR spectra of MBA (top), the product (middle), and DMMA (bottom) (the labelled is the unreacted DMMA).

^{13}C NMR of MBA (100 MHz, DMSO-d_6): 51.4, 36.3, 31.6, 20.0, 14.0.

^{13}C NMR of the product (100 MHz, DMSO-d_6): 171.6, 171.5, 168.1, 167.9, 142.1, 141.7, 124.7, 124.2, 49.1, 45.2, 34.7, 30.8, 29.4, 28.1, 19.6, 19.5, 18.2, 17.7, 14.0, 13.9, 13.9. 13.7.

^{13}C NMR of DMMA (100 MHz, DMSO-d_6): 166.5, 140.4, 9.2.

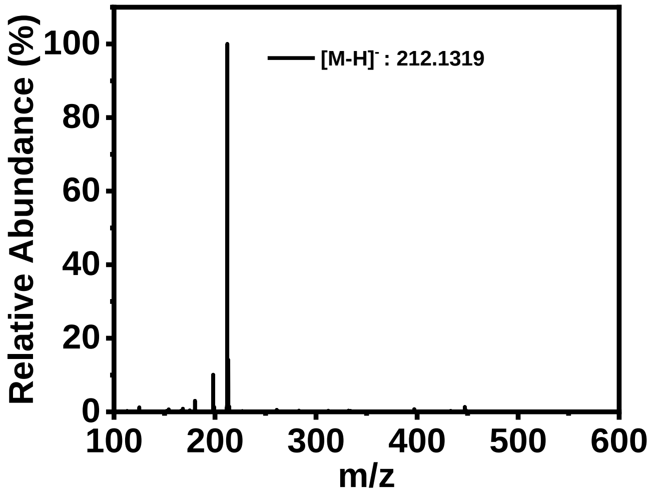

Supplementary Figure 2. ESI-MS spectrum of the product of the reaction between DMMA and MBA. The mass peak with a found m/z for [M-H]$^-$ is close to its calculated m/z of 212.1292.

2.2 Determination of the reaction rate constant of the reaction between DMMA and MBA

Kinetic studies were performed with DMMA and MBA at a constant temperature, and were monitored by UV-vis spectroscopy. In this system, the peak around 283 nm (Abs@283 nm) is ascribed to the difference value of the absorption between the anhydride ring and product, thus Abs@283 nm was used to monitor the kinetics of the reaction in anhydrous DMSO at 20 °C.

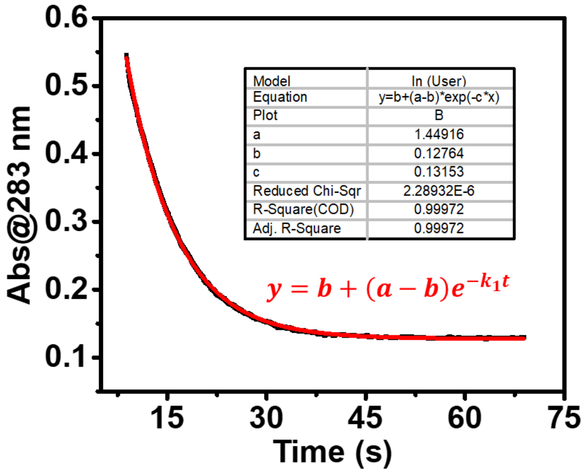

Supplementary Figure 3. Diagram of Abs@283 versus reaction time of 10 mM MBA and 0.5 mM DMMA at 20 °C and the fitting curves (red).

On the one hand, the kinetic of the reaction for DMMA in the presence of 20 eq of MBA was explored through UV-vis spectroscopy. Firstly, 200 μL 100 mM MBA was added into 1790 μL DMSO. After that, 10 μL 100 mM DMMA was added into the above solution and shaken quickly, then put into the detection cell. The Abs@283 nm was recorded with the time going at 20°C. Through the fitting as pseudo first-order reaction kinetics in the presence of 20 eq of MBA, the relationship between Abs@283 nm and time (t) follows:

$$Abs@283\ nm = b + (a - b)e^{-k_1 t}$$

where a and b are constants, and k_1 represents the pseudo first-order reaction rate constant and is 0.13 s^{-1} at 20 °C (Figure S3).

On the other hand, the kinetic of the reaction for DMMA and equivalent MBA was also explored in the same method. Firstly, 20 μL 100 mM MBA was added into 1960 μL DMSO. After that, 20 μL 100 mM DMMA was added into the above solution and shaken quickly, then put into the detection cell. The Abs@283 nm was recorded with the time going at 20 °C. Through the fitting as second-order reaction kinetics, the relationship between Abs@283 nm and time (t) follows:

$$Abs@283\ nm = b + \frac{a - b}{k_2 t + 1}$$

where a and b are constants, and k_2 represents the second-order reaction rate constant and is 6.29 M^{-1} s^{-1} at 20 °C.

2.3 Cleavage of the addition product into protonated MBA and DMMA

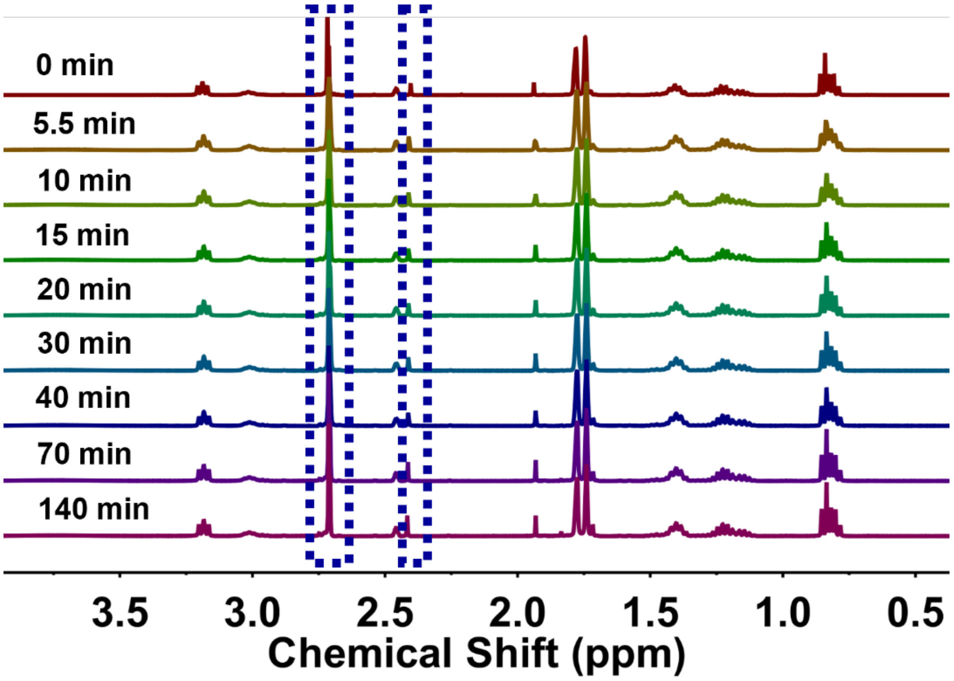

Supplementary Figure 4. ^{1}H NMR spectra (400 MHz) of the product in DMSO-d_6 with varied time after adding D_2O.

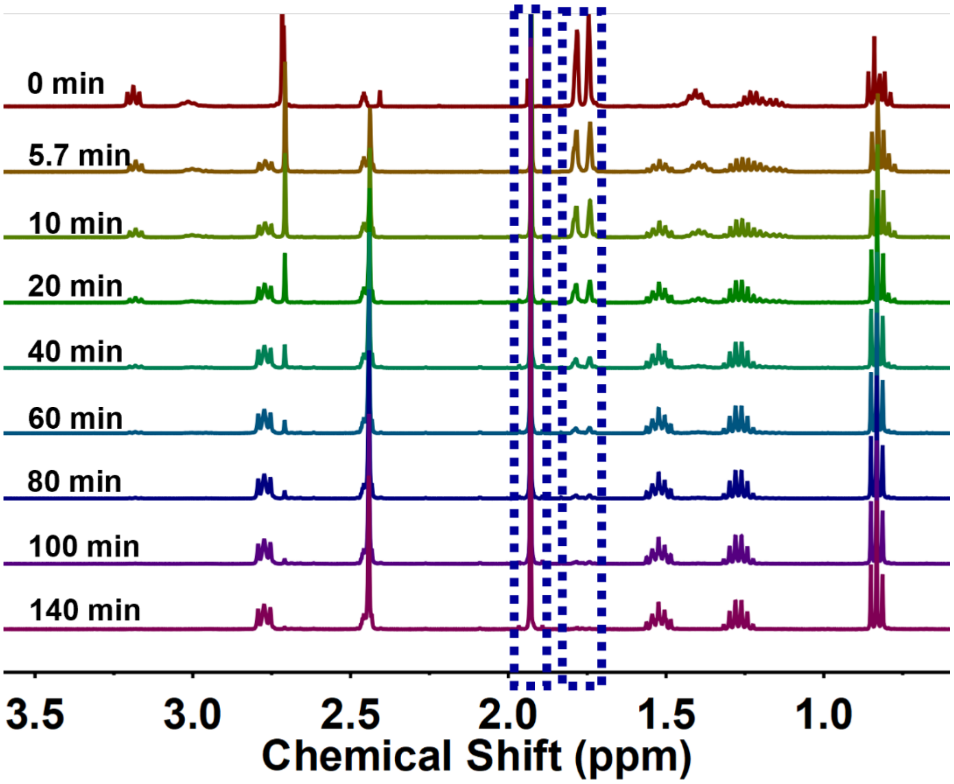

Supplementary Figure 5. ^{1}H NMR spectra (400 MHz) of the product in DMSO-d_6 with varied time after adding DCl/D_2O.

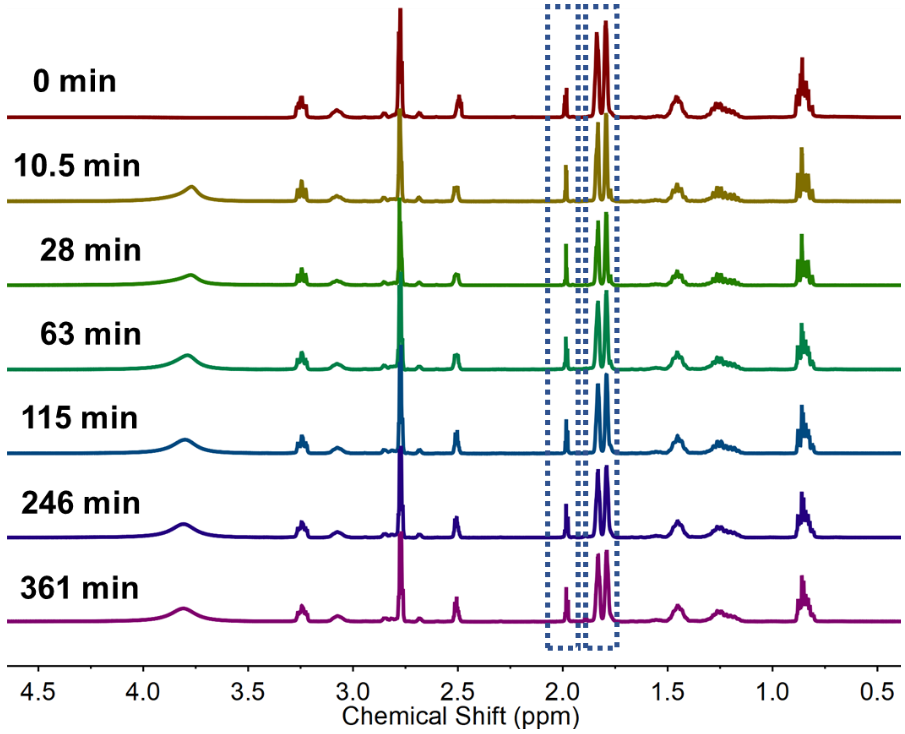

Supplementary Figure 6. ^{1}H NMR (400 MHz) spectra of the product at 0 °C in the mixture of DMSO-d_6 and DMF-d_7 with varied time after adding D_2O.

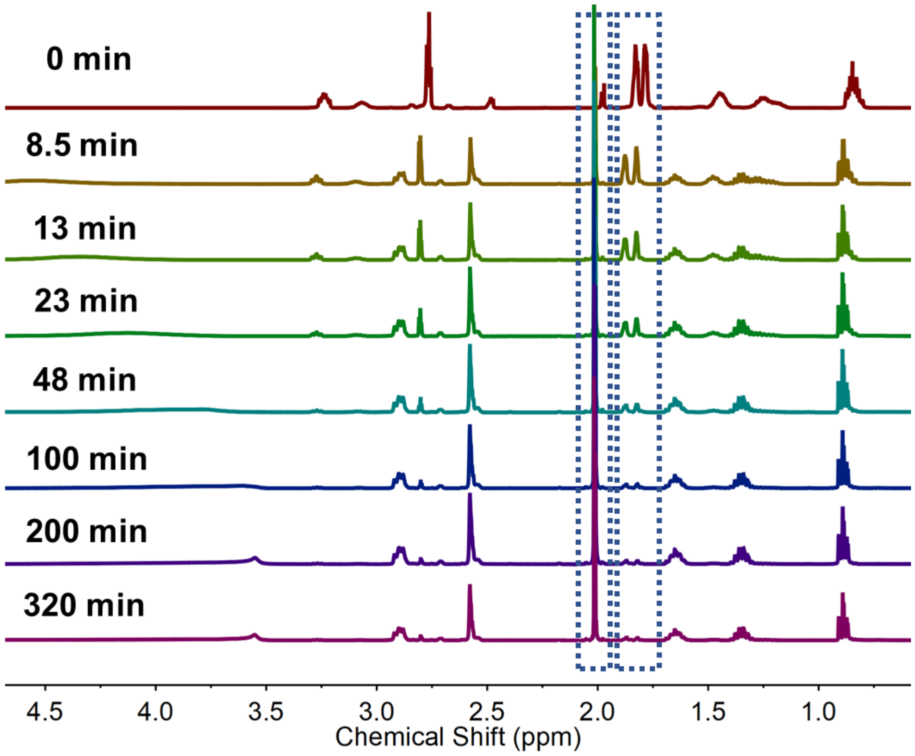

Supplementary Figure 7. ^{1}H NMR spectra (400 MHz) of the product at 0 °C in the mixture of DMSO-d_6 and DMF-d_7 with varied time after adding DCl/D_2O.

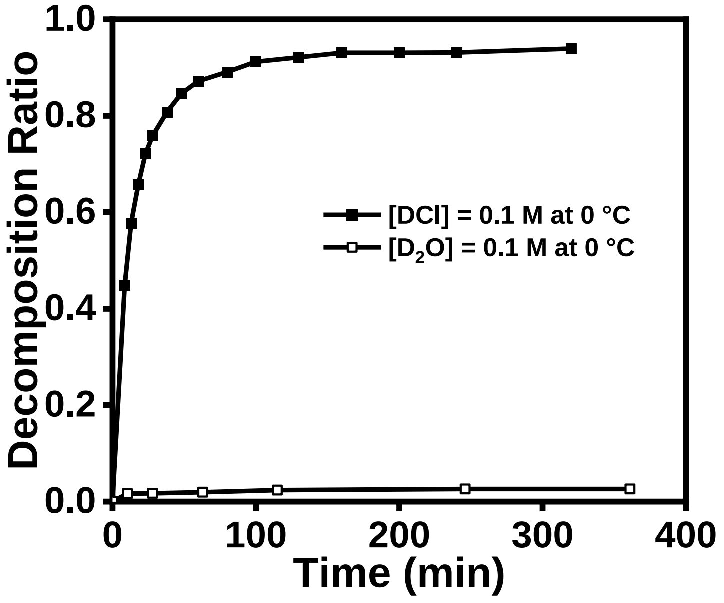

Supplementary Figure 8. Plot of the cleavage ratio versus the reaction time with 0.1 M DCl and D_2O at 0 °C in the mixture of DMSO-d_6 and DMF-d_7, respectively.

The cleavage reaction of the addition product into protonated MBA and DMMA under acid conditions was monitored through time-dependent NMR spectroscopy. Firstly, a solution of the product with 100 mM was prepared in DMSO-d_6. Then, DCl aqueous solution (20 wt%) was added into the above solution to make the concentration of DCl as 0.1 M. The ^{1}H NMR spectra were recorded with certain interval time. In this system, the cleavage ratio was calculated on the basis of the following equation:

$$Cleavage\ Ratio = \left[\left(\frac{\delta_{1.82}+\delta_{1.79}}{\delta_{1.98}+\delta_{1.82}+\delta_{1.79}}\right)_0 - \left(\frac{\delta_{1.82}+\delta_{1.79}}{\delta_{1.98}+\delta_{1.82}+\delta_{1.79}}\right)_t\right] \times 100\%$$

The cleavage reaction under neutral conditions was also monitored through the same way by adding the same volume of D_2O. In this system, to obtain the accurate integration of peaks, the cleavage ratio was calculated on the basis of the following equation:

$$Cleavage\ Ratio = \left[\left(\frac{\delta_{2.76}}{\delta_{2.76}+\delta_{2.44}}\right)_0 - \left(\frac{\delta_{2.76}}{\delta_{2.76}+\delta_{2.44}}\right)_t\right] \times 100\%$$

The cleavage reaction in the mixture of DMSO-d_6 and DMF-d_7 at 0 °C was monitored through time-dependent NMR spectroscopy in the same way.

3. Synthesis and Characterization of Bifunctional Maleic Anhydride Monomer (BMA)

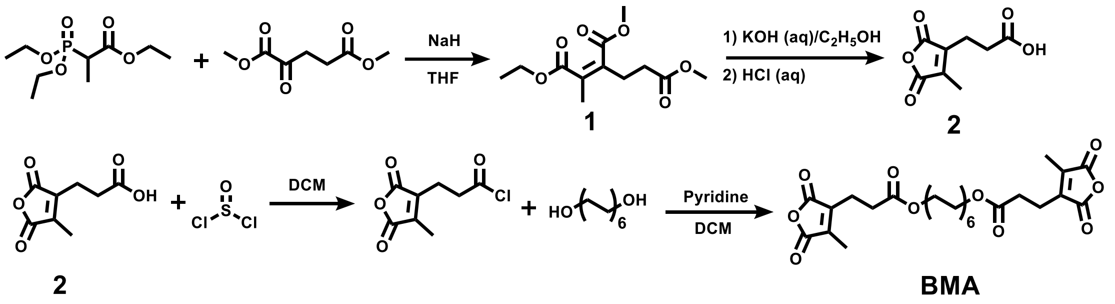

Supplementary Figure 9. Synthetic route of bifunctional maleic anhydride monomer BMA

The synthetic routes of compound **1** and **2** are modified according to the previous reference.[1] Sodium hydride (3.46 g, 86.5 mmol, 60% w/w in mineral oil) was suspended in 170 mL anhydrous THF in the ice-water bath. Triethyl-2-phosphonopropionate (25.0 g, 105 mmol) was added dropwise into the above suspension in the ice-water bath, and the mixture was stirred for 10 min until hydrogen gas generated entirely. Then dimethyl-2-oxoglutarate (12.05 g, 69.2 mmol) was added and the mixture was stirred for 30 min in the ice-water bath. Then, saturated ammonium chloride aqueous solution (265 mL) was added into the reaction solution, and the mixture was extracted with diethyl ether. Afterwards, the mixture was purified by silica gel column chromatography (petroleum ether : ethyl acetate = 4:1 V/V) to obtain compound **1** (14.6 g, 82% yield). ^{1}H NMR (400 MHz, $CDCl_3$): δ (ppm): 4.22 (q, J = 7.2 Hz, 2H), 3.75 (s, 3H), 3.68 (s, 3H), 2.67 (t, J = 8.0 Hz, 2H), 2.48 (t, J = 8.4 Hz 2H), 2.00 (s, 3H), 1.29 (t, J = 7.2 Hz, 3H).

Compound **1** (14.64 g, 56.7 mmol) was dissolved in the mixture of ethanol (180 mL) and 2 M KOH (aq, 100 mL). Then, the solution was stirred at 100 °C for 1 h and turned yellow. After cooling to room temperature, the solution was acidified with 1 M HCl aqueous solution (200 mL) to tune pH = 1, and then extracted with ethyl acetate (250 mL). Finally, the solvent was evaporated to gain compound **2** without further purification (9.59 g, 92% yield). ^{1}H NMR (400 MHz, $CDCl_3$): δ (ppm): 2.77 (s, 4H), 2.12 (s, 3H).

Compound **2** (9.59 g, 52.1 mmol) was dissolved in 25 mL anhydrous CH_2Cl_2. Then, 38.6 mL sulfoxide chloride was added into the above solution, after which the solution was stirred at 40 °C for 12 h. After vacuum evaporation to remove the excess sulfoxide chloride and solvents, 65 mL anhydrous CH_2Cl_2 was added to dissolve the product. Then, 1,12-dodecanediol (4.80 g, 23.7 mmol) and pyridine (2.3 mL) were added into the above solution under the ice-water bath. The reaction went for 6 h at room temperature. The mixture was extracted with saturated ammonium chloride aqueous solution (70 mL × 2). The organic phase was dried by Na_2SO_4. Finally, the product was purified by silica gel column chromatography (petroleum ether : ethyl acetate = 4:1 V/V) to afford pale yellow solid **BMA** (7.8 g, 62% yield). ^{1}H NMR (400 MHz, $CDCl_3$): δ (ppm): 4.02 (t, J = 6.8 Hz, 2H), 2.65-2.71 (m, 4H), 2.09 (s, 3H), 1.56 (m, 2H), 1.22 (br, 8H). ^{13}C NMR (100 MHz, $CDCl_3$): 171.8, 166.0, 165.7, 142.4, 142.2, 65.3, 31.1, 29.5, 29.5, 29.3, 28.6, 25.9, 20.0, 9.7. ESI-MS: founded $[BMA+NH_4]^+$ = 552.2780, calculated $[BMA+NH_4]^+$ = 552.2803.

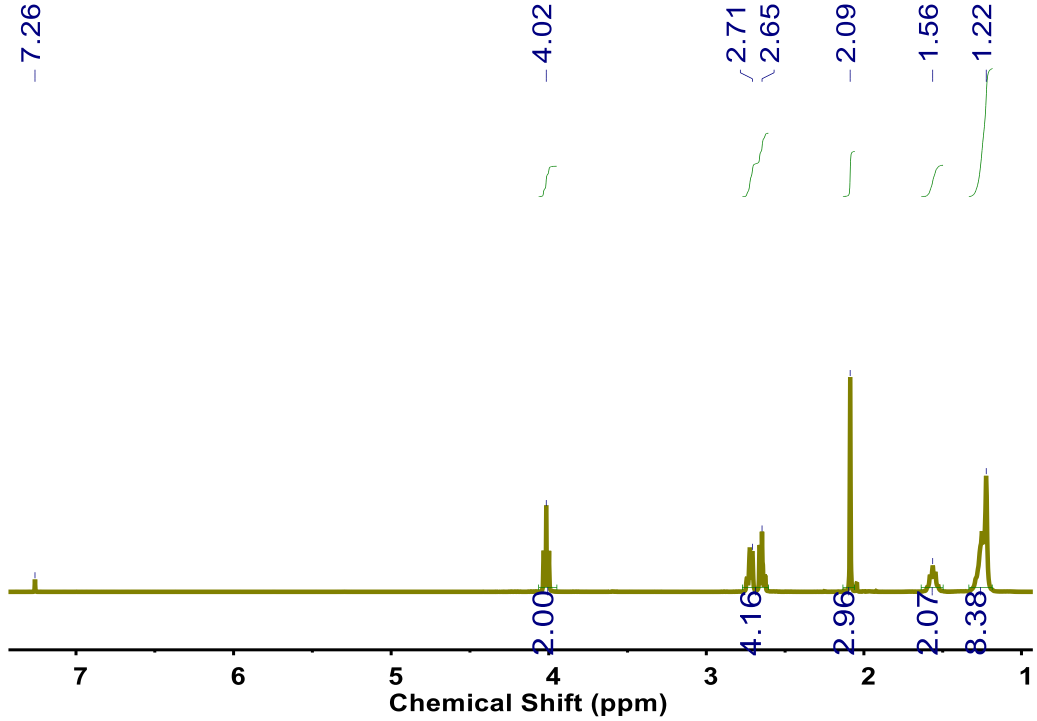

Supplementary Figure 10. ^{1}H NMR Spectrum (400 MHz) of anhydride monomer BMA in $CDCl_3$.

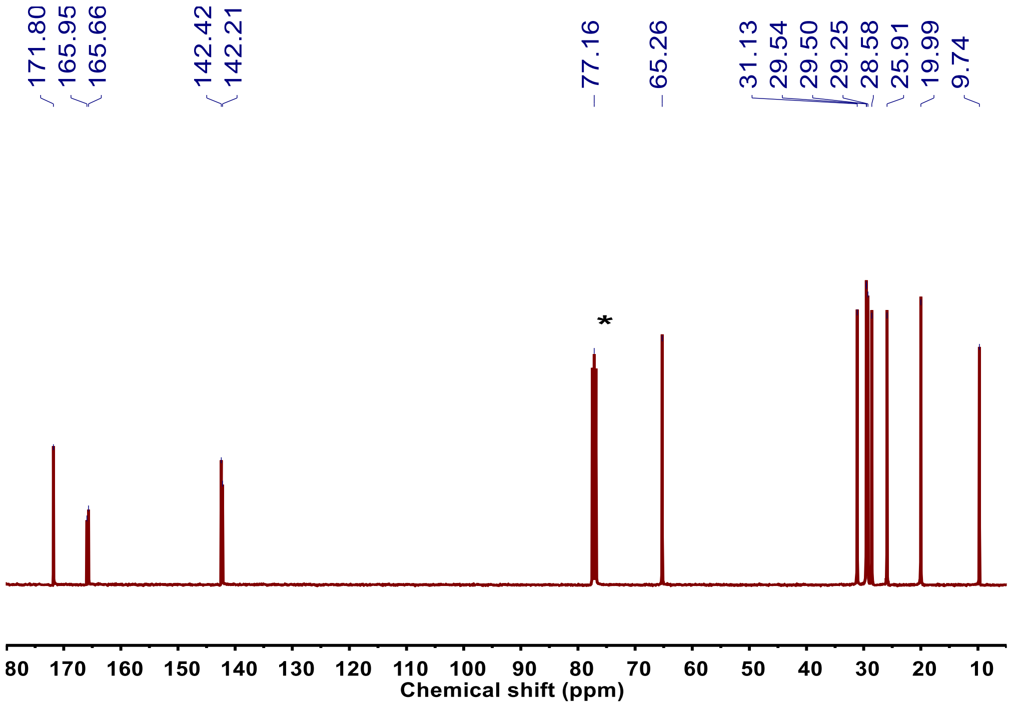

Supplementary Figure 11. ^{13}C NMR Spectrum (100 MHz) of anhydride monomer BMA in $CDCl_3$.

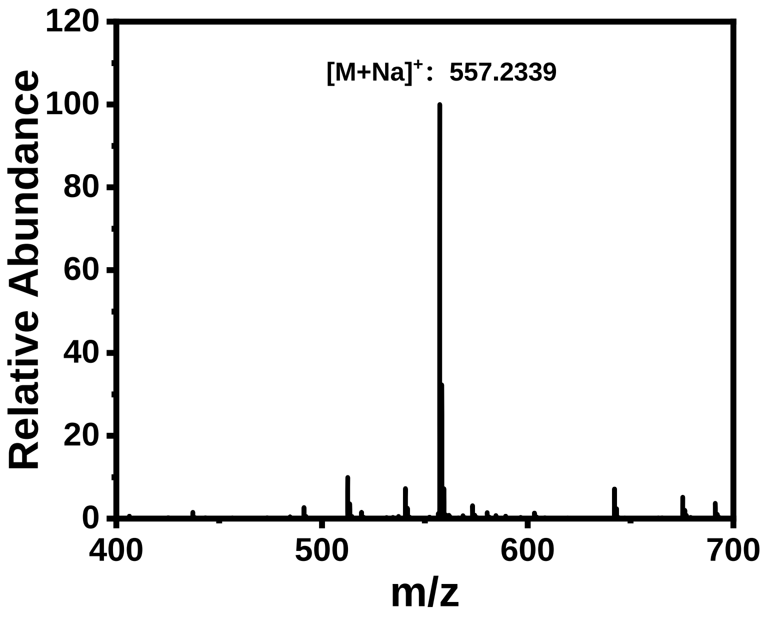

Supplementary Figure 12. ESI-MS spectrum of BMA. The mass peak with a found m/z for $[M+Na]^+$ is close to its calculated m/z of 557.2357.

4. Characterization of the Amidation Reaction between BMA and Bifunctional Secondary Amines

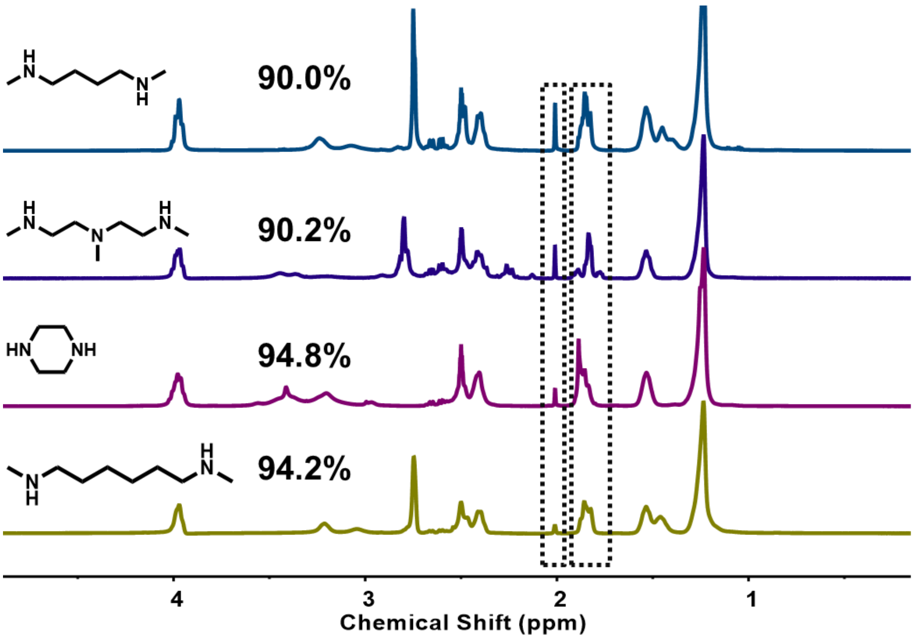

Supplementary Figure 13. ^{1}HNMR spectra (400 MHz) of the addition product of bifunctional monomer BMA and bifunctional secondary amines, and the corresponding conversion.

5. Synthesis and Characterization of Polyamic Acid Networks BMA-TMEN

5.1 Photograph of the polyamic acid network BMA-TMEN

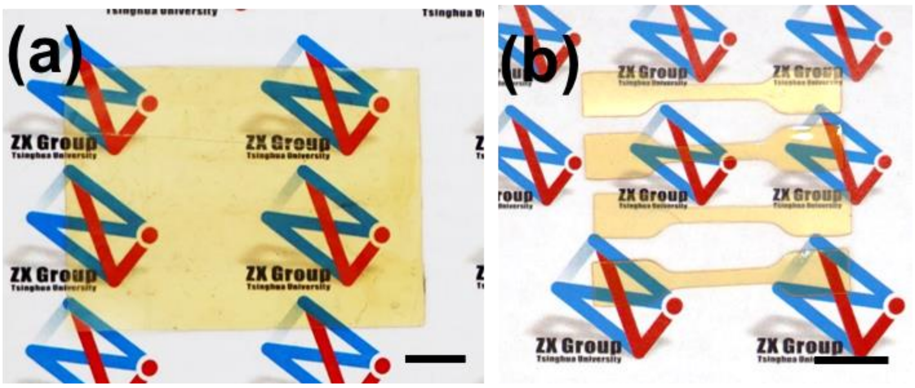

Supplementary Figure 14. Photograph of (a) the polymer network BMA-TMEN and (b) the dog-bone BMA-TMEN samples. (Scale bar: 1 cm)

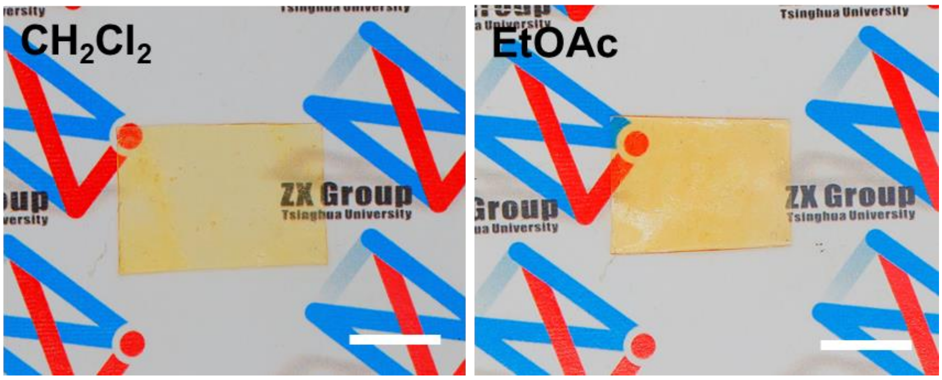

Supplementary Figure 15. Photograph of the polymer network BMA-TMEN prepared from CH_2Cl_2 (left) and ethyl acetate (right). (Scale bar: 1 cm)

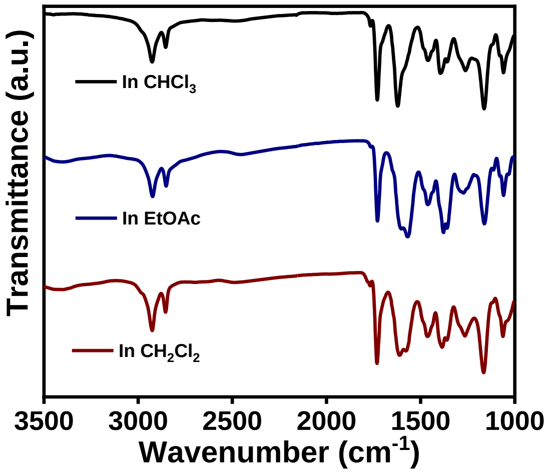

Supplementary Figure 16. FT-IR spectra of BMA-TMEN prepared from $CHCl_3$, CH_2Cl_2, and ethyl acetate.

5.2 DSC and DMA of the polyamic acid network BMA-TMEN

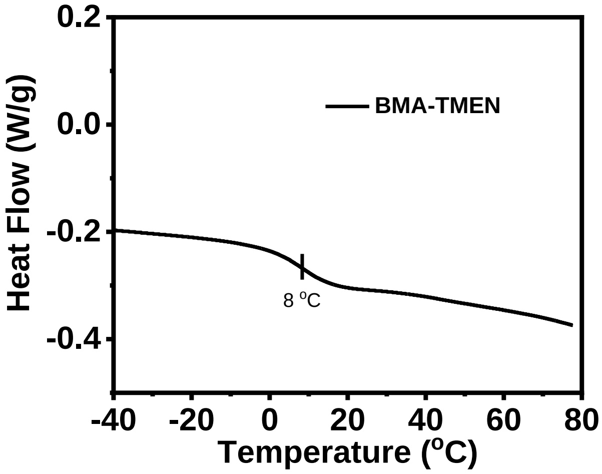

Supplementary Figure 17. DSC curve of BMA-TMEN

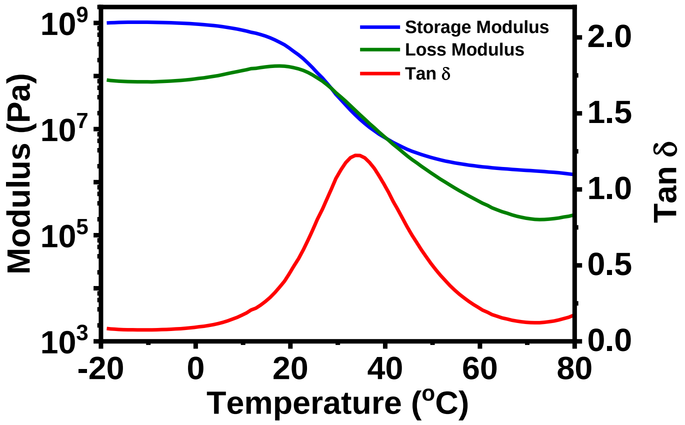

Supplementary Figure 18. DMA curve of BMA-TMEN

6. Chemical Recycling and Reconstruction of Polyamic Acid Networks

BMA-TMEN

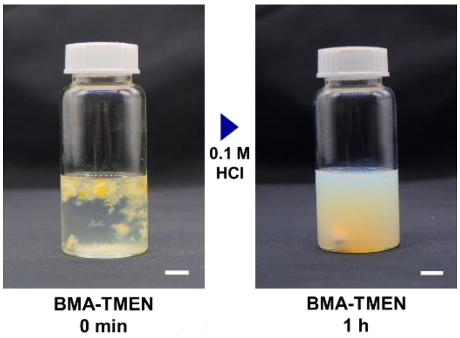

Supplementary Figure 19. Photograph of the depolymerization of BMA-TMEN networks in 0.1 M HCl. (Scale bar: 1 cm)

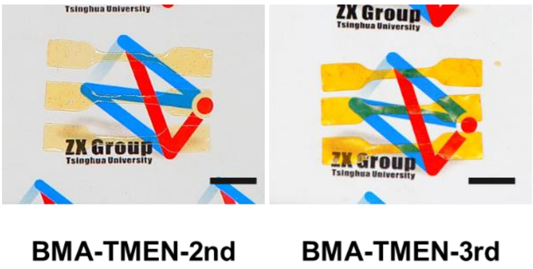

Supplementary Figure 20. Photograph of the repolymerized polymer network BMA-TMEN (Scale bar: 1 cm)

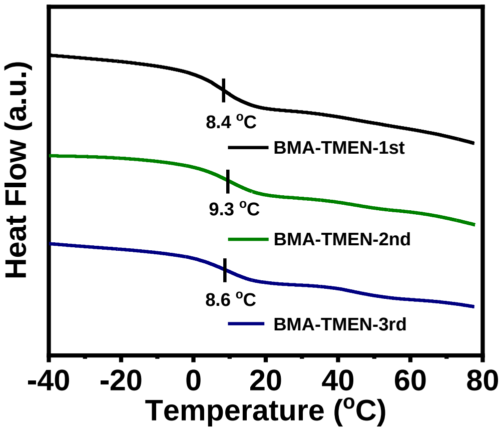

Supplementary Figure 21. DSC curves of BMA-TMEN and repolymerized BMA-TMEN from recycled monomers.

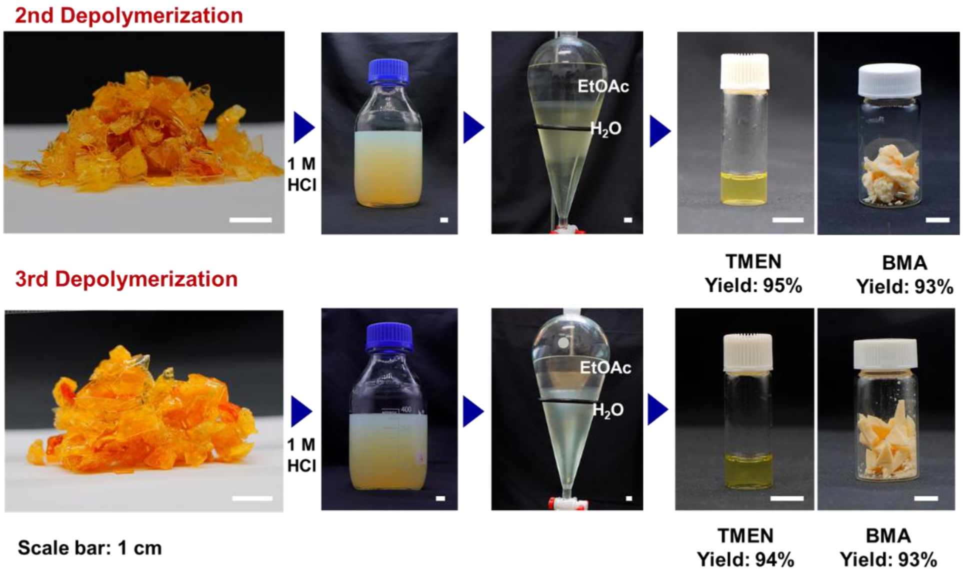

Supplementary Figure 22. Photographs of the multiple depolymerization of polymer networks, the separation and recycling procedures of BMA and TMEN monomers by the liquid-liquid extraction method (scale bar: 1 cm).

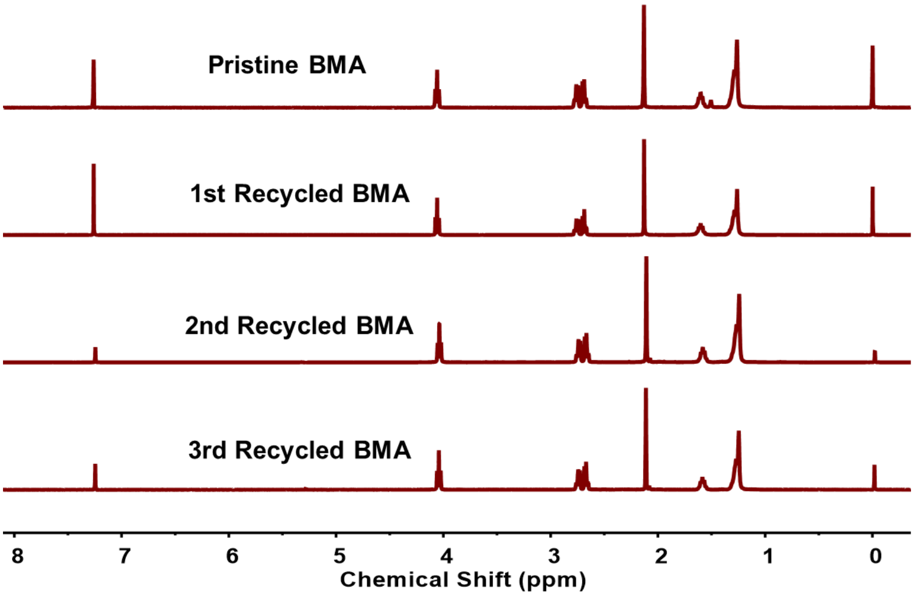

Supplementary Figure 23. ^{1}H NMR spectra (400 MHz) of pristine and recycled BMA.

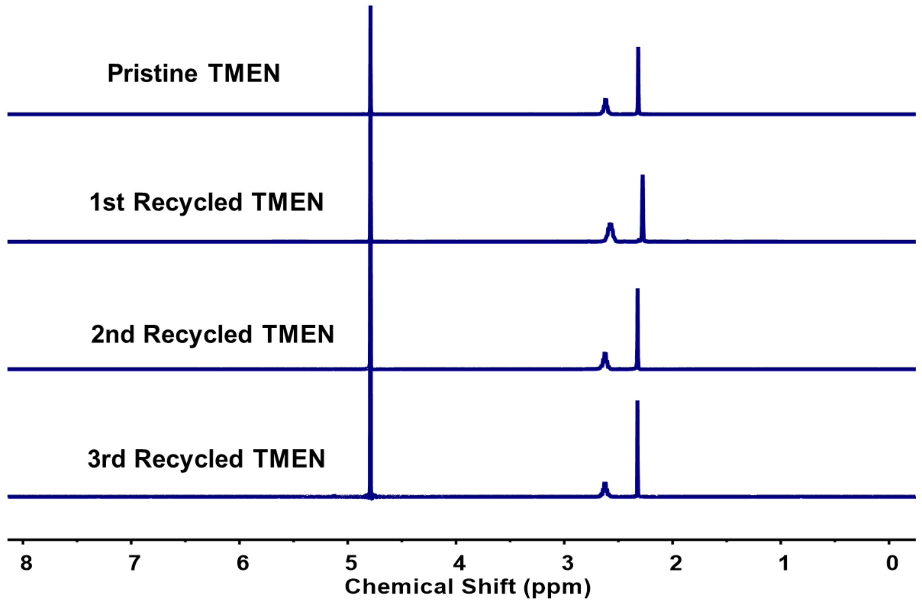

Supplementary Figure 24. ^{1}H NMR spectra (400 MHz) of pristine and recycled TMEN.

7. Synthesis and Characterization of Linear Polyethyleneimine (LPEI)

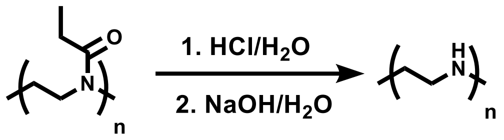

Supplementary Figure 25. Synthetic route of LPEI.

The synthetic procedures of LPEI were modified according to the previous reference.[2] Poly(2-ethyl-2-oxazoline) (10.0 g, M_w ~ 200 kDa) was dissolved in 52 mL water and 82 mL concentrated HCl aqueous solution. Then, the solution was stirred at 100 °C for 12 h. After cooling to room temperature, the white precipitate was filtered and washed with 15 mL water. Then, 2 M NaOH was added to dissolve the residue until pH = 10 and all the solid was dissolved at 85 °C. The viscous solution was cooled to room temperature, producing a large number of white precipitates. The white precipitate was filtered and washed with 50 mL water and then freeze-dried to afford white solid **LPEI** (4.1 g, 94% yield). ^{1}H NMR (400 MHz, D_2O): δ (ppm): 2.74 (br, N-CH_2).

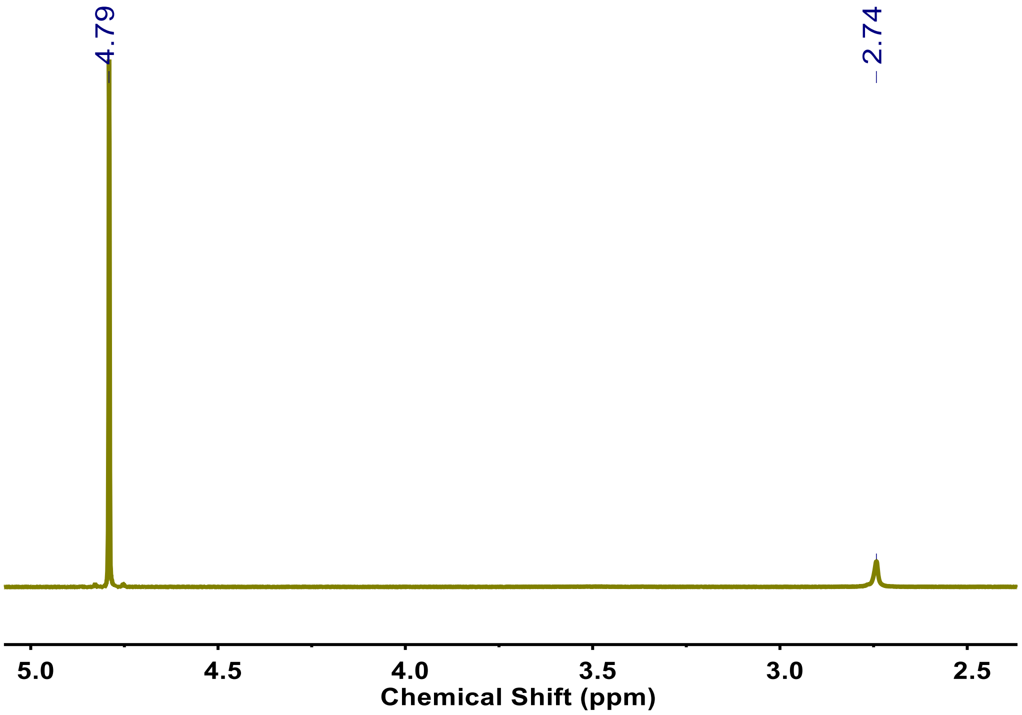

Supplementary Figure 26. ^1H NMR Spectrum (400 MHz) of LPEI in D_2O.

8. Synthesis and Characterization of Polyamic Acid Networks LPEI-BMA$_x$

8.1 Synthetic procedures of Polymer networks LPEI-BMA$_x$

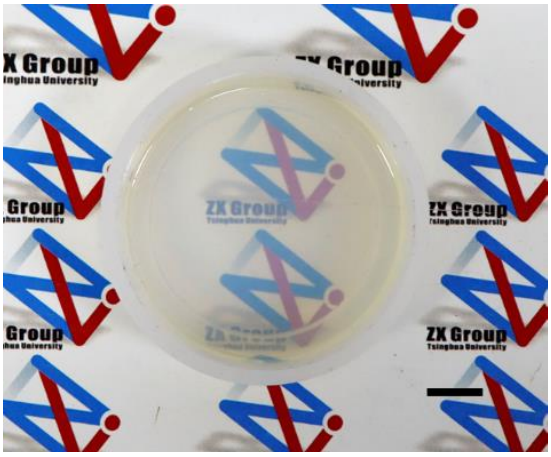

Supplementary Figure 27. Photograph of the polymer network LPEI-BMA$_{0.10}$. (Scale bar: 1 cm)

8.2 FT-IR spectra of LPEI-BMA$_x$

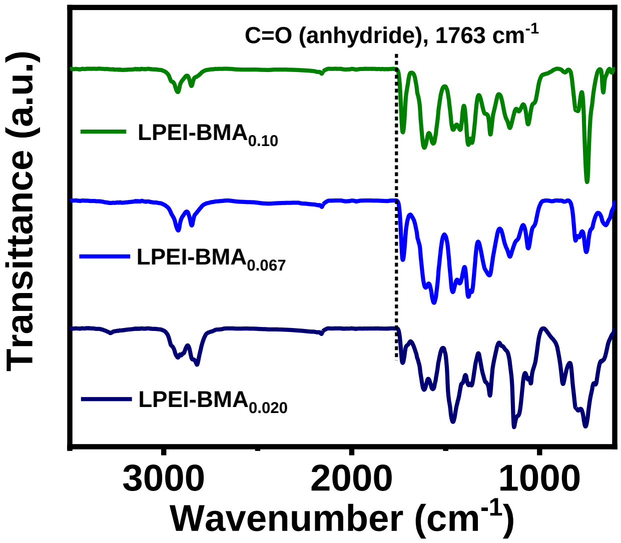

Supplementary Figure 28. FT-IR spectra of LPEI-BMA$_{0.10}$, LPEI-BMA$_{0.067}$, LPEI-BMA$_{0.020}$.

Through the FT-IR spectra, the peak at 1763 cm^{-1} ascribed to the C=O vibration in the anhydride unit is not observed, indicating the high conversion of BMA in polyamic acid networks LPEI-BMA$_x$.

8.3 Mechanical and thermal properties of LPEI-BMA$_x$

Supplementary Table 1. The mechanical properties and T_g of LPEI-BMA$_x$.

	Breaking strength (MPa)	Breaking elongation (%)	Young's modulus (MPa)	Yield Stress (MPa)	T_g[a] (°C)	T_g[b] (°C)
**LPEI-BMA$_{0.020}$**	3.0±0.22	413±11	2.5±0.31	--	-5.6	1.7
**LPEI-BMA$_{0.050}$**	8.2±1.67	483±32	21.4±0.99	--	18.7	17.2
**LPEI-BMA$_{0.067}$**	31.9±2.11	370±12	269±16	--	27.7	30.6
**LPEI-BMA$_{0.10}$**	32.1±0.65	118±6	880±19	38.3±3.3	53.4	48.9

a: determined by DSC; b: determined by the fitting the initial drop in storage modulus measured by DMA.

9. Chemical Recycling and Reconstruction of Polyamic Acid Networks

LPEI-BMA$_{0.10}$

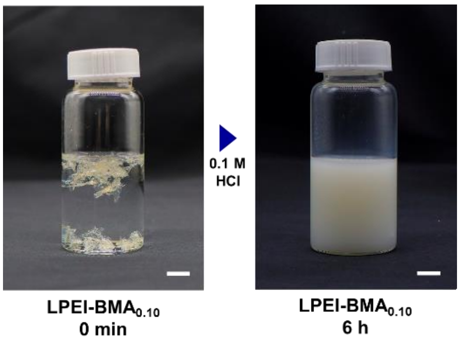

Supplementary Figure 29. Photograph of the depolymerization of LPEI-BMA$_{0.10}$ networks in 0.1 M HCl. (Scale bar: 1 cm)

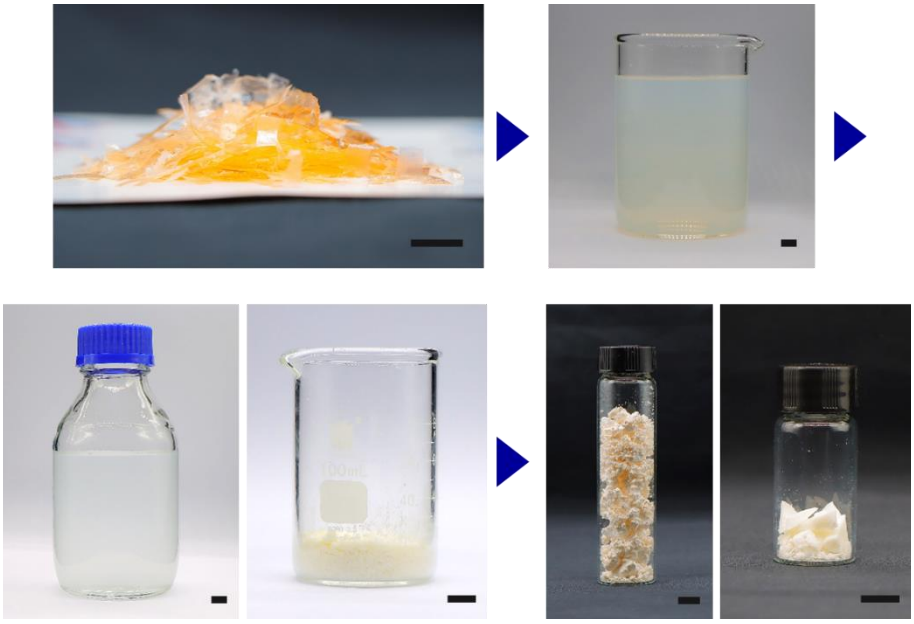

Supplementary Figure 30. Photographs of the 1st depolymerization of polymer networks, the separation and recycling procedures of BMA and LPEI by the precipitation method (scale bar: 1 cm).

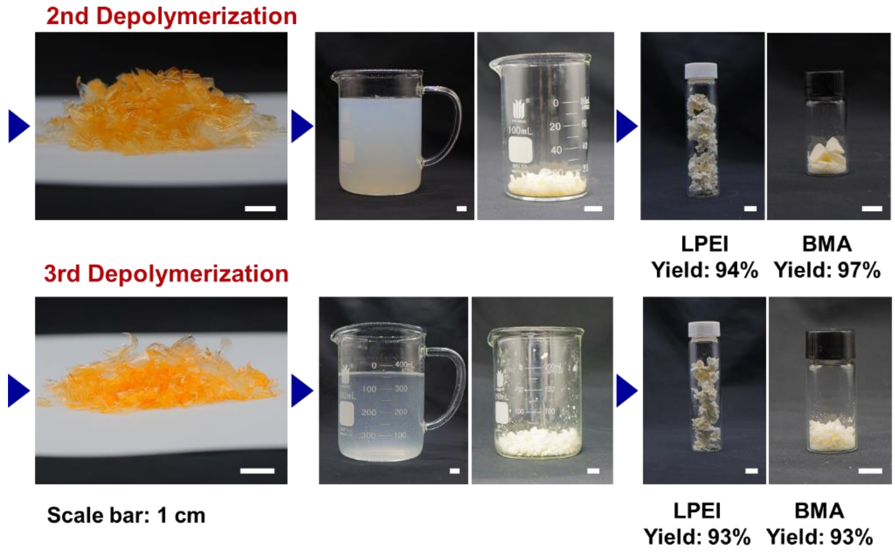

Supplementary Figure 31. Photographs of the 2nd and 3rd depolymerization of polymer networks by the repolymerization of recycled monomers, the separation and recycling procedures of BMA and LPEI (scale bar: 1 cm).

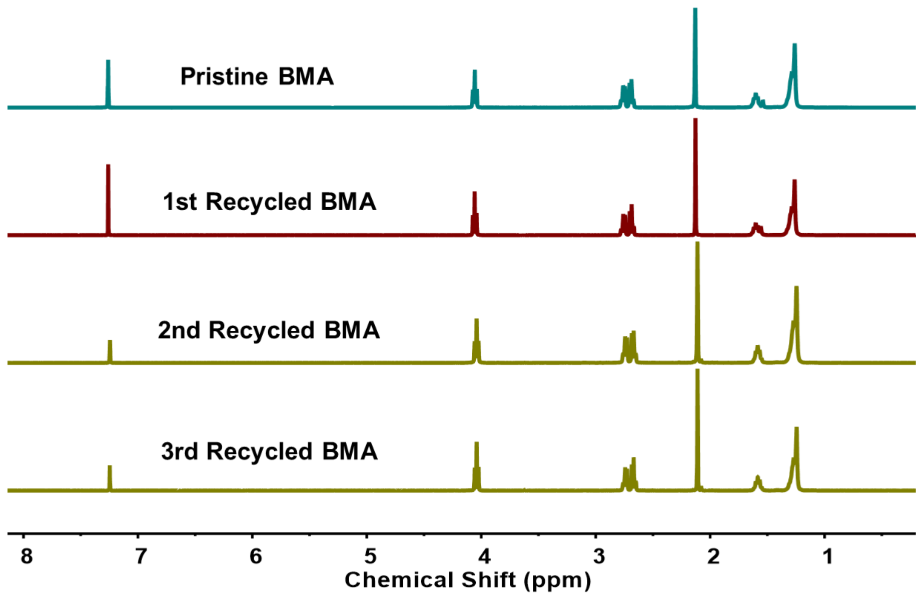

Supplementary Figure 32. ^{1}H NMR spectra (400 MHz) of pristine and recycled BMA.

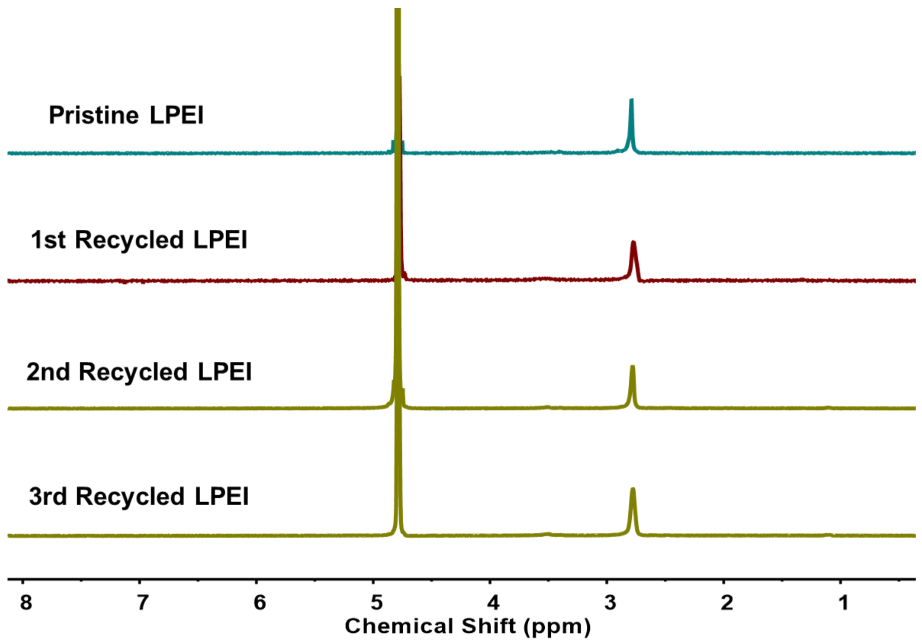

Supplementary Figure 33. ^1H NMR spectra (400 MHz) of pristine and recycled LPEI.

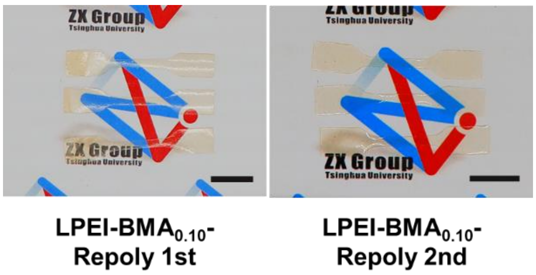

Supplementary Figure 34. Photograph of the repolymerized polymer network LPEI-$BMA_{0.10}$ (Scale bar: 1 cm)

10. Supplementary References:

[1] J. Yang, Z. Yin, Y. Chang, H. Wang, J.-F. Xu, X. Zhang. Giant, 2021, 6, 100052.

[2] L. Tauhardt, K. Kempe, K. Knop, E. Altuntas,, M. Jager, S. Schubert, D. Fischer, U. S. Schubert. Macromol. Chem. Phys., 2011, 212, 1918.
